# Automated selection of homologs to track the evolutionary history of proteins

**DOI:** 10.1186/s12859-018-2457-y

**Published:** 2018-11-19

**Authors:** Pablo Mier, Antonio J. Pérez-Pulido, Miguel A. Andrade-Navarro

**Affiliations:** 1 Faculty of Biology, Johannes Gutenberg University Mainz, Hans-Dieter-Hüsch-Weg 15, 55128 Mainz, Germany; 2 Universidad Pablo de Olavide, Sevilla, Spain

**Keywords:** Homology, Web tool, Evolutionary path

## Abstract

**Background:**

The selection of distant homologs of a query protein under study is a usual and useful application of protein sequence databases. Such sets of homologs are often applied to investigate the function of a protein and the degree to which experimental results can be transferred from one organism to another. In particular, a variety of databases facilitates static browsing for orthologs. However, these resources have a limited power when identifying orthologs between taxonomically distant species. In addition, in some situations, for a given query protein, it is advantageous to compare the sets of orthologs from different specific organisms: this recursive step-wise search might give an idea of the evolutionary path of the protein as a series of consecutive steps, for example gaining or losing domains. However, a step-wise orthology search is a time-consuming task if the number of steps is high.

**Results:**

To illustrate a solution for this problem, we present the web tool ProteinPathTracker, which allows to track the evolutionary history of a query protein by locating homologs in selected proteomes along several evolutionary paths. Additional functionalities include locking a region of interest to follow its evolution in the discovered homologous sequences and the study of the protein function evolution by analysis of the annotations of the homologs.

**Conclusions:**

ProteinPathTracker is an easy-to-use web tool that automatises the practice of looking for selected homologs in distant species in a straightforward way for non-expert users.

**Electronic supplementary material:**

The online version of this article (10.1186/s12859-018-2457-y) contains supplementary material, which is available to authorized users.

## Background

Homologous protein sequences are those with a common evolutionary origin. There are two types of protein homologs, depending on how they originated: paralogs, derived from a gene duplication event, and orthologs, originated from a speciation event [1]. The ortholog conjecture postulates that orthologous sequences are functionally more similar than paralogous sequences in comparable divergence times [2–4]. This assumption has been proven in several studies [5–7], but it is yet to be widely accepted [3, 8]. In molecular biology research, and particularly in the field of comparative genomics, the ortholog conjecture is central in the functional annotation of genes and proteins. It implies that given an experimentally annotated protein sequence, its functional properties and sequence features are assumed to be shared by its orthologs. Thus, the correct identification of orthologous sequences is a key process in the automatic inference of protein function.

A plethora of computational tools has been developed to identify orthologs from a broad spectrum of model and non-model organisms. These methods can be either graph-based, in which graphs are built with sequences as nodes and edges as similarity scores obtained after a BLAST similarity search, or depend on phylogenies, which analyse trees to identify evolutionary events [9, 10]. Furthermore, several orthology databases offer precomputed sets of orthologs [11–16]. These resources are very useful and accurate for focused queries. Here, we want to propose a different, augmented, more flexible approach to the use of homology for function investigation, which is not covered by these static databases of orthologs, and which we find necessary and useful.

**Fig. 1:**
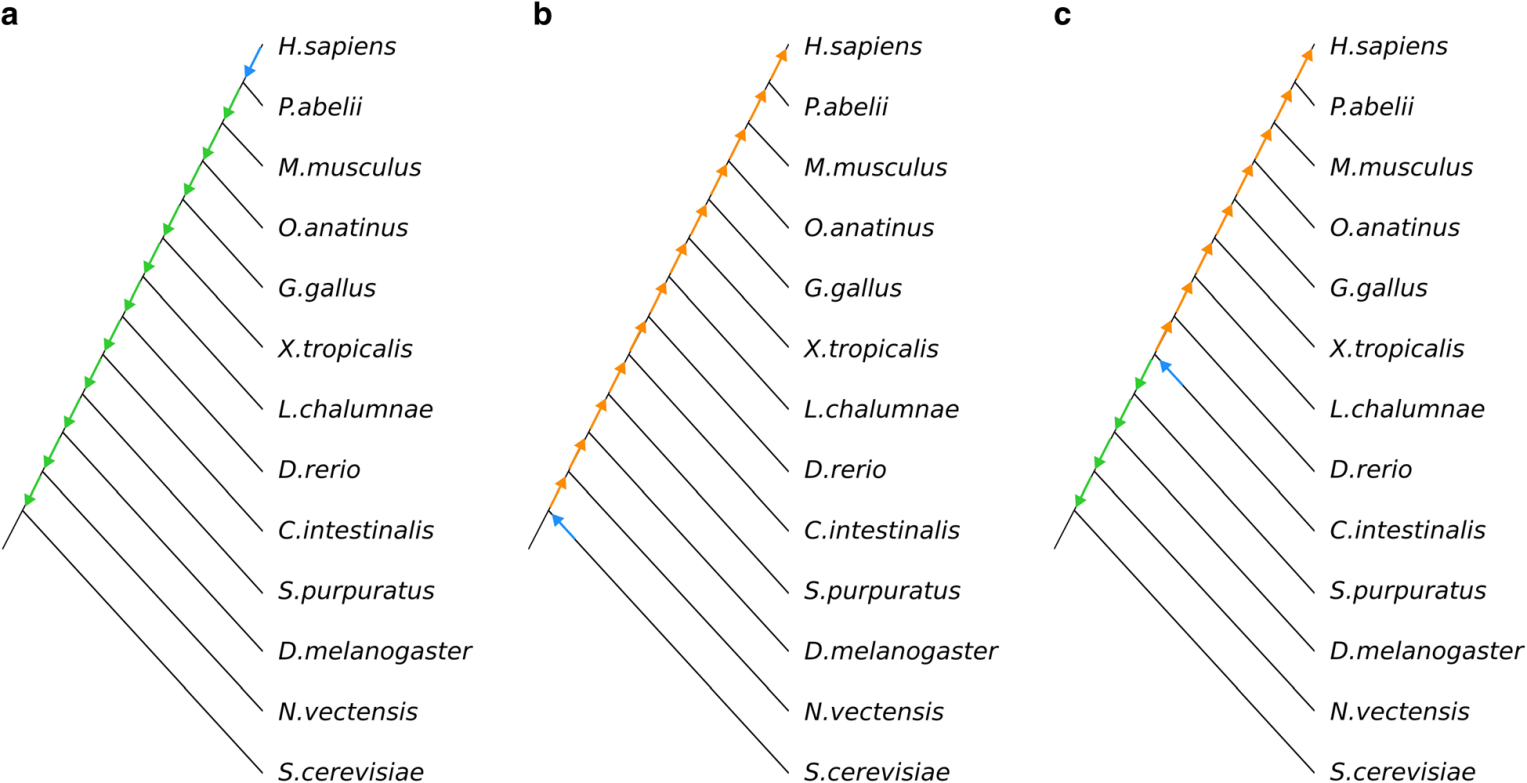
Simplification of a step-wise search strategy to locate homologs at increasingly distant taxonomic divisions from human. Assuming we want to study the evolution of a protein family from a common ancestor of human and yeast, towards a human protein member of this family, we can collect selected homologs in three ways (see text for details). From **a**) human to *S. cerevisiae*, if we start from a human protein, **b**) from *S. cerevisiae* to human, if we start from a yeast protein, and **c**) from an intermediate species such as *C. intestinalis* both to human and *S. cerevisiae*. The color of the arrows represents either the starting species for the search (blue), or whether the direction of the search is towards closer (orange) or further (green) taxonomic divisions to human

Given a query protein family and a species of interest, we might be interested to know the step-wise evolution of proteins of this family throughout the species tree towards the species of interest. This can be used to infer the evolution of its sequence and to relate it to the evolution of its function. Considering human as the species of interest, if we have a human member of this protein family, we could obtain and compare homologs of this protein in species at increasingly distant taxonomic divisions englobing the central species. These groups of homologs will suggest a series of common ancestors at increasing evolutionary distances. For example, if we are interested in a human protein, we could look for a homolog in chimpanzee (non-human primate), then we could use this homolog to search in mouse (non-primate eutherian), and the mouse homolog to search in platypus (non-eutherian mammal), and so on until we find no homolog (Fig. 1a). This collection of homologs of a query sequence from multiple increasingly distant taxonomic divisions, can help to understand the evolution of the features of the query protein, their order of emergence, and their evolutionary context.

If we have a protein of this family in a very distant species and we are not sure about the corresponding human homolog protein, we can try the step-wise search strategy presented above in reverse, searching for homologs of the protein in the distant species at increasingly closer taxonomic distances to the central species. For example, given a yeast protein, if we want to trace the evolution of the common ancestral protein between human and yeast to human, we could search for the yeast homolog in a taxonomic division closer to human, for example in *Nematostella vectensis* (non-bilaterian metazoan). We could have gone directly for a human homolog. But, having the homolog in *Nematostella* can inform us of the properties on the metazoan ancestral protein. Then, we could search for a homolog of the *Nematostella* sequence in *Drosophila melanogaster* (non-deuterostomian bilaterian), use the *Drosophila* homolog to search in *Strongylocentrotus purpuratus* (non-chordata deuterostomian), the *S. purpuratus* homolog to search in *Ciona intestinalis* (non-Osteichthyes chordata), and so on until eventually closing on human (Fig. 1b).

In fact, we could start this procedure with a species giving us an intermediate point of the path (e.g. a protein in *C. intestinalis*) and then proceed with iterative processes in each direction (Fig. 1c). In this example, towards both closer (*C. intestinalis* > *Danio rerio* > … > Human) and further (*C. intestinalis* > *S. purpuratus* > *…* > *S. cerevisiae*) taxonomic divisions to human.

Such step-wise approach requires to find the closest homolog of a sequence in another species. The Reciprocal Best Hits BLAST (RBHB) approach [17] is usually used to find this homolog and assess whether it is an ortholog or not. The identification of homologs (and orthologs) would be ideally performed by RBHB in a supervised manner, controlling setting parameters and assessing the evolutionary relationship of two or more sequences after an individual examination of the search reports. The evident drawbacks are that it is a time-consuming procedure and that it vastly depends on the user knowledge about the search process and its specific computational parameters.

In the exploratory procedure described above, there is the additional complexity that one might want to try different paths, or different species (e.g. we took *Drosophila* as non-deurostomian bilaterian, but another insect such as the mosquito *Anopheles gambiae* would do, or, for that matter, the worm *Caenorhabditis elegans*). We would as well like to skip steps in which a homolog is not found in a species, because it might be that this given species lacks a detectable member of the family due to orthologous displacement or errors in the sequencing of the corresponding genome. In addition, it is desirable to impose restrictions in the search to try to focus the set of selected homologs on a chain of orthologs. Thus, if in one of the steps in the chain of step-by-step consecutive searches an ortholog is not found, then the search on the next step will be done with the last ortholog found. This ensures that all sequences selected as orthologs are connected in a chain, but at the same time keeps non-orthologs that might be informative. Each of these changes and procedures would take a large effort as new species-to-species searches would need to be combined, compared and stored.

To facilitate this type of evolutionary analysis, we developed ProteinPathTracker, a web server that helps assessing the evolutionary history of proteins by automatically performing sequential RBHB along an evolutionary path, which can be selected by the user. It is an automation of the manual process of identification of orthologs through distant species, designed to be fast and easy to use by non-expert users. ProteinPathTracker also includes two additional optional functionalities, regarding the study of the evolution of a subsequence in the identified homologs, and the evolution of functional annotations of proteins.

## Results

### Implementing an automatic strategy for iterative homology searches

Orthology is usually assessed by performing RBHB [17]. This procedure relies on a double sequence similarity search, to check the assumed principle that orthologs are the most similar sequences in two proteomes [1, 4]. But mutation rates are not linear in evolution and vary between genomic loci [18–20]. This implies that similarity searches may not be always enough to determine orthologs when comparing distant species. Some methods have been developed to discover distant homologs, such as the PSI-BLAST algorithm, which searches for new homologs starting from those found in previous search steps [21]. But these methods lack of standardisation and they strongly depend on drifts in the databases.

Here we propose an alternative iterative search for orthologs that splits the search in subsequent steps, using proteomes from model organisms to perform RBHB searches repeatedly, and uses the orthologous sequence found in one search as the query for the next one. This step-wise alternative provides an ordered series of orthologs that can be used to infer an evolutionary path in terms of ancestral sequences. It imposes a more constrained search for orthologs than a direct search between taxonomically distant species, which can help to select functionally relevant homologs (see Discussion).

**Fig. 2:**
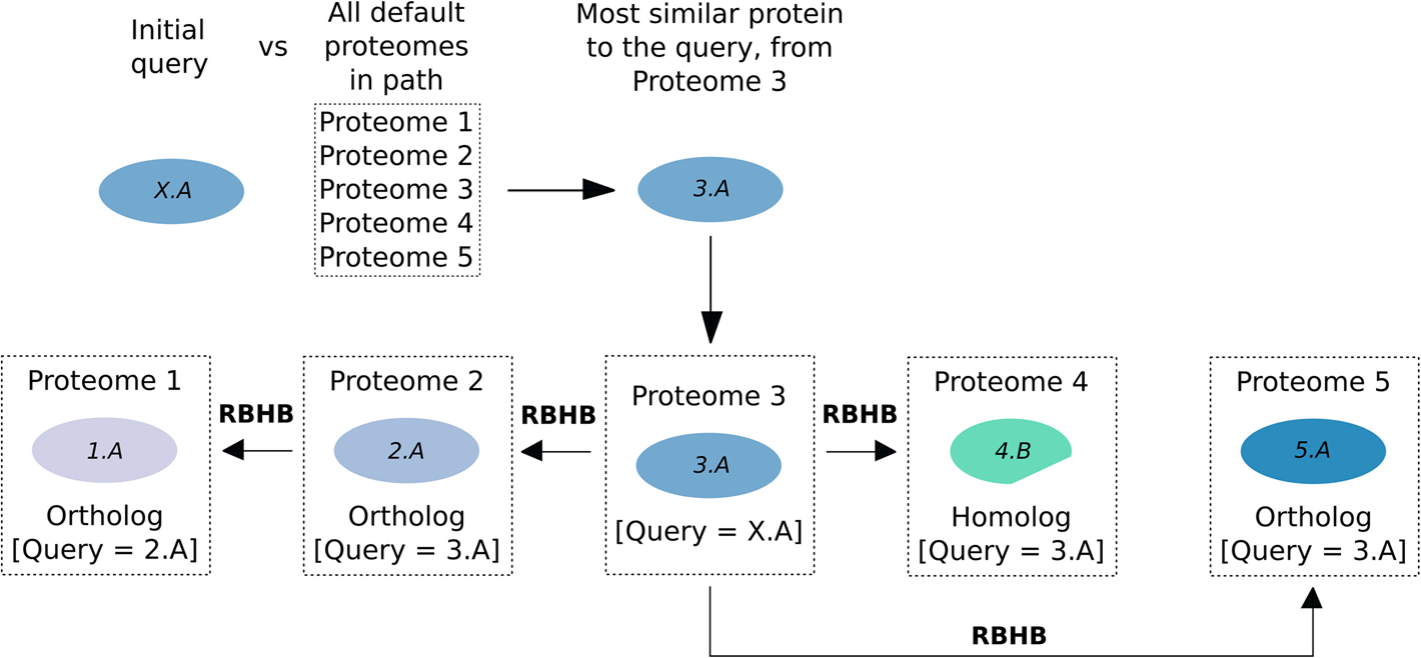
Schematic pipeline of ProteinPathTracker. A path composed of five proteomes is used as example, and the protein *X.A* as the initial query. RBHB: Reciprocal Best Hits BLAST

To automatise the proposed step-by-step homology search we created the ProteinPathTracker web tool [22]. Given a query protein and a selected evolutionary path, ProteinPathTracker performs a homology search to look for the most similar protein to the query in a database composed by all the default proteomes of the selected path (Fig. 2). Homology searches are performed using the BLAST 2.2.31 package [21] with default parameters. Using the found protein to get in the proteome path (taxon *X*), ProteinPathTracker looks for its closest homolog in the previous proteome (taxon *X-1*). If this is an ortholog, then, the identified ortholog from the proteome *X-1* is used to look for its closest homolog in the next proteome *X-2*, etc. The same strategy is followed from proteome *X* to proteome *X + 1*, then *X + 2*, and so on. The procedure is repeated until all the selected proteomes in the evolutionary path are covered. If orthology cannot be assured after one search, the hit is annotated as homolog. In that case, the last ortholog found in the evolutionary path is again used to look for homologs in the next proteome.

The default evolutionary path covers the main taxonomic groups (14 intermediate taxa) between cellular organisms and the genus Homo, with the ultimate purpose of tracking the orthologs of human proteins in bacterial or archaeal model organisms. One proteome is selected by default per taxon, but up to 193 complete reference proteomes can be selected by the user (Additional file 1). Furthermore, there are five additional evolutionary paths focused on some of the most used model organisms that the user can select to track the evolution of a protein: 1) from Primates to Homo, 2) from Viridiplantae to Arabidopsis, 3) from Fungi to Schizosaccharomyces, 4) from Bacteria to Escherichia and 5) from Arthropoda to Drosophila. Each proteome is placed in the taxon which is the last one shared with the terminal node of the path.

### Searching for orthologous sequences in distant species

**Fig. 3:**
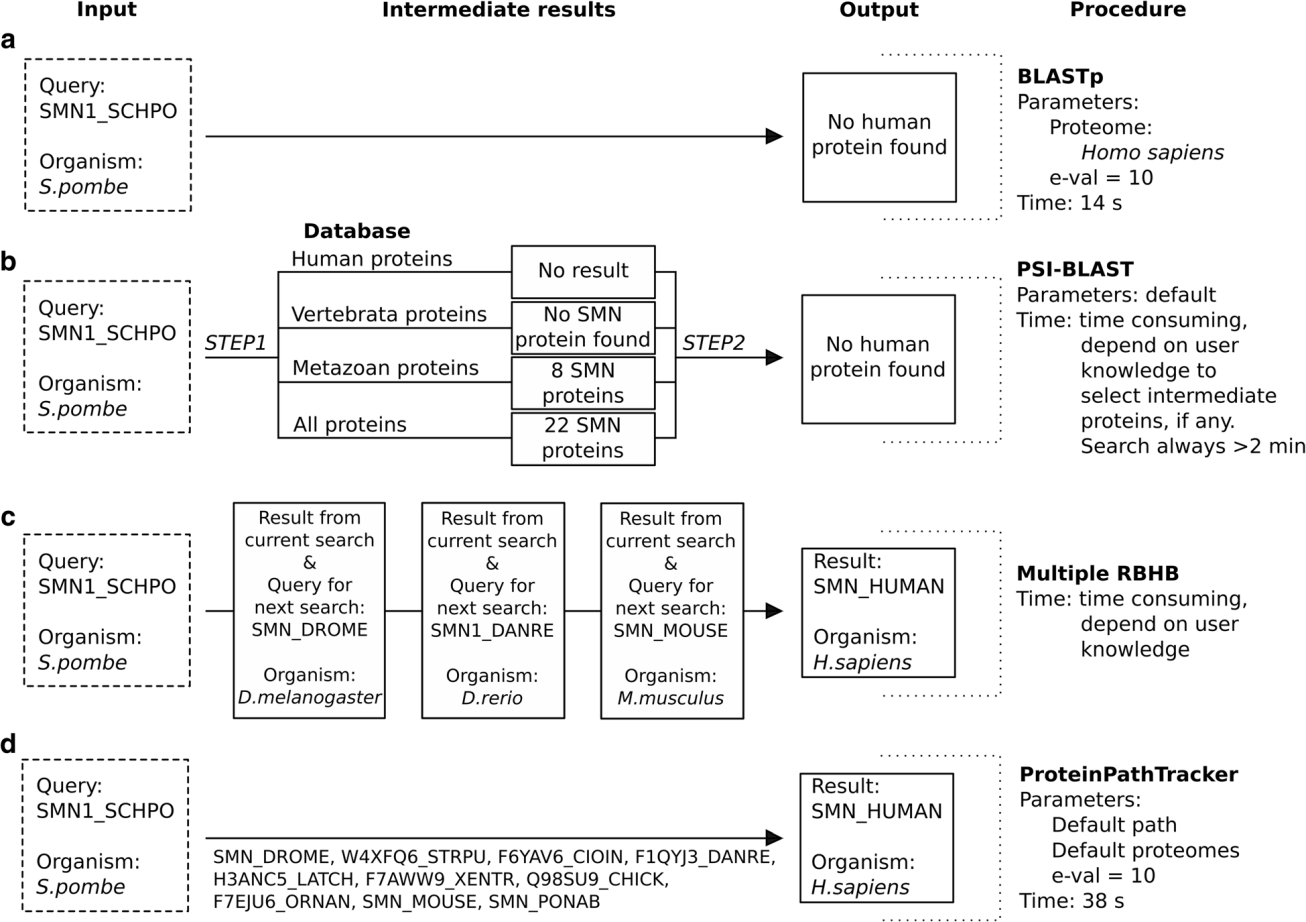
Case study of a search to locate the human ortholog of the protein SMN1_SCHPO. The procedure, intermediate results and output of four strategies are shown: **a**) BLASTp, **b**) PSI-BLAST, **c**) Multiple RBHB, **d**) ProteinPathTracker

As a case study, we used the protein SMN1, which is present in fungal organisms, invertebrates and vertebrates but has a poorly conserved protein sequence [23]. When performing a BLASTp search of the fission yeast *Schizosaccharomyces pombe* Smn1 protein (UniProt:SMN1_SCHPO) with default parameters at the NCBI BLAST site, it is not possible to discover any orthologous sequences in *Homo sapiens* (Fig. 3a). A similar result is obtained when using the most thorough PSI-BLAST algorithm (Fig. 3b), even if using several subsets of the non-redundant protein database available from the NCBI.

But if one manually looks for the ortholog of the query sequence SMN1_SCHPO in *Drosophila melanogaster* (fruit fly), and the procedure is subsequently repeated in the proteomes from *Danio rerio* (zebrafish) and *Mus musculus* (mouse), a human orthologous protein is found (Fig. 3c). This process is automated in ProteinPathTracker (Fig. 3d), as described in the previous section. All sequences considered as intermediate orthologs from SMN1_SCHPO to SMN_HUMAN in the result obtained from the ProteinPathTracker execution conserve sequence features corresponding unequivocally with SMN proteins [23]. The presented example is a clear case in which ProteinPathTracker allows in a fast and easy manner to track the evolutionary path of a query protein.

### Tracking the evolutionary conservation of residues in protein motifs

ProteinPathTracker includes an optional functionality to ‘lock a region’. It allows the reconstruction of the evolutionary history of a protein motif, by mapping it in the discovered homologs. Different to other methods that use specific sequence motifs to improve the homology discovery [24], ProteinPathTraker first looks for the homologs of a query sequence and then locates the motif within them, as opposed to directly performing the search for the motif in the database.

Locking a functional annotated subsequence helps in assessing whether the homologs harbor the motif, and even when it appeared in evolution. ProteinPathTracker allows the inspection of locked regions selected by homolog, coordinates of the region in the sequence, and minimum coverage required in the other aligned sequences. Since these analyses are applied to the ProteinPathTracker results, the examination of different locked regions is direct as no new sequence searches or alignments are necessary.

**Fig. 4:**
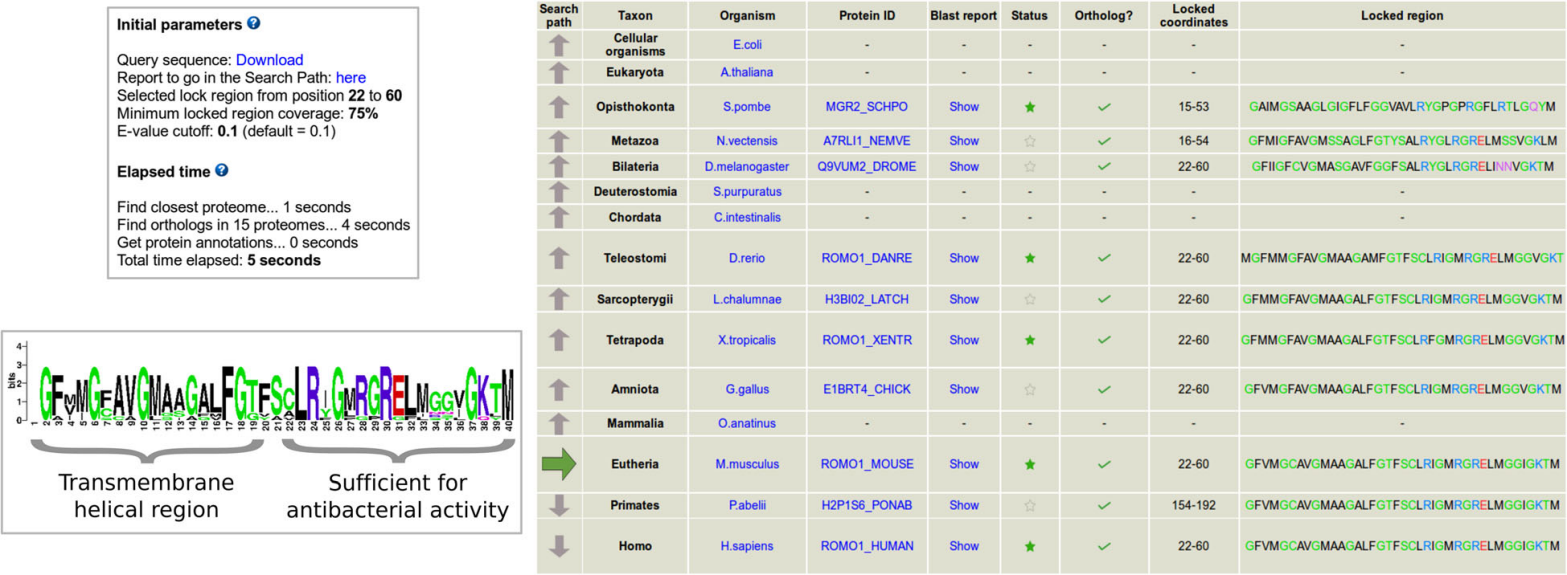
Results obtained after the execution of ProteinPathTracker to illustrate the locking region functionality. The protein ROMO1_MOUSE was used as the query sequence in a search in the default evolutionary path with the default proteomes. The coordinates 22–60 were locked in the query

To illustrate the use of this feature, we run ProteinPathTracker with ROMO1_MOUSE (UniProt:P60603), a Reactive oxygen species modulator from the Romo1/Mgr2 protein family, using default path and proteomes, and then locked the subsequence 22–60 in this protein (Fig. 4). That subsequence includes two overlapping positional annotations in the query protein: a transmembrane helical region (coordinates 22–44), and a region sufficient for antibacterial activity (coordinates 42–60). Orthologous sequences to the query protein were found in proteomes from the fission yeast to human, in a very short time. Furthermore, the subsequence was locked in all the results. The logo built out of them clearly shows the two separate regions within. First, an N-terminal region (corresponding to the annotated transmembrane region) characterised by five glycines separated by three residues each (GxxxGxxxG). This is consistent with two glycine zippers known to be important in transmembrane helices [25]. Second, a region annotated to be involved in antibacterial activity, with a conserved LRxGxRGR motif.

### Prevalence of GO terms in human homologs

Tracking functional annotations from selected homologs can be useful to study the evolution and emergence of new functions. ProteinPathTracker offers functional annotation of the discovered homologs, which it is of interest to perform functional assignments. To systematically evaluate the performance of ProteinPathTracker in this respect, we executed it using all human proteins as individual queries, and we studied the prevalence of annotation terms in the sets of selected homologs at different evolutionary distances, starting from human. For every query, we computed the presence of orthologs and homologs in the default proteomes and obtained their GO terms (see Methods for details).

**Fig. 5:**
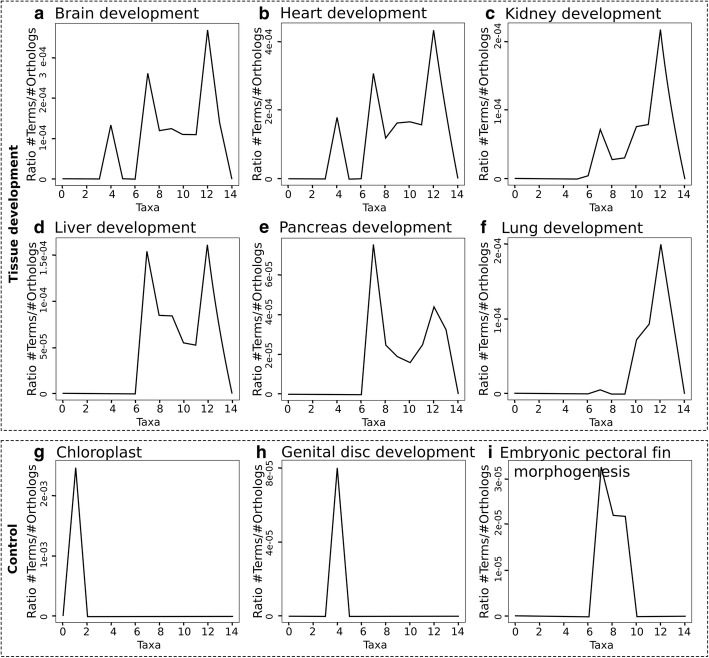
Distribution of the ratio of specific GO terms per taxon in selected homologs of human proteins. Fifteen taxa were considered: 0) cellular organisms, 1) Eukaryota, 2) Opisthokonta, 3) Metazoa, 4) Bilateria, 5) Deuterostomia, 6) Chordata, 7) Osteichthyes, 8) Sarcopterygii, 9) Tetrapoda, 10) Amniota, 11) Mammalia, 12) Eutheria, 13) Primates, 14) Homo. Plots do not show any value for taxon 14 (Homo), as human proteins were used as query sequences. One panel per GO term: **a**) brain development, **b**) heart development, **c**) kidney development, **d**) liver development, **e**) pancreas development, **f**) lung development, **g**) chloroplast, **h**) genital disc development, **i**) embryonic pectoral fin morphogenesis

The examination of the distribution of individual GO terms shows the evolutionary relationship between the discovered sequences. Here we discuss some GO terms related to organ development, as their emergence in evolution is well described in the literature. The distribution of homologs of human proteins annotated with the GO terms *brain development* and *heart development* are similar, with a first peak in Bilateria (taxon = 4, animals with bilateral symmetry) and a spread after Osteichthyes (taxon = 7, jawed vertebrates) (Fig. 5a-b). The early evolution of the brain is supported by studies suggesting that glial cells evolved in the last common ancestor of Protostomia and Deuterostomia [26, 27], which in our study would be in Bilateria (taxon = 4), and by their complexity in zebrafish (taxon = 7) [28]. Similarly, primitive cardiac myocytes first appeared in Bilateria [29]. On the other hand, proteins related to the development of other organs such as kidney, liver and pancreas seem to have evolutionarily appeared in Osteichthyes (Fig. 5c-e). Interestingly, while proteins related to *liver development* appear from taxon 7, the ones annotated with the more complex process of *liver regeneration* do not appear until taxon 10 (data not shown). Of all human complex organs, lungs would be one of the most modern ones, absent until Amniota (taxon = 10) (Fig. 5f) [30].

As a control, we studied the distribution of a plant-specific term (*chloroplast*), a *Drosophila*-specific term (*genital disc development*) and a term specific to finned organisms (*embryonic pectoral fin morphogenesis*) (Fig. 5g-i). The three of them are restricted to specific taxa, as expected: *chloroplast* to taxon 1 (Eukaryota), with the plants; *genital disc development* to taxon 4 (Bilateria), where *Drosophila* species are; and *embryonic pectoral fin morphogenesis* to taxa 7–9 (Osteichthyes-Tetrapoda), covering finned species from zebrafish to frogs.

The GO annotations of the results from ProteinPathTracker are provided as a table of GO annotations obtained from all the homologs of the query sequence. For comparison, we also provide the possibility of examining the graphs presented in Fig. 5 for each GO term. This allows the user to assess whether the distribution of a given GO term found in the human homologs reflects the behavior of most human homologs. For example, a researcher may wonder if the reason for not finding homologs with the GO term *pancreas development* for a given human protein in levels 4 to 8 is due to no homolog in these levels having that function. In retrieving the graph (Fig. 5e), the researcher will see that there are indeed homologs to human proteins associated to pancreas development in levels 7 and 8. Then it can be concluded that the homologs in level 7 and 8 could have been potentially associated to this function. It remains unknown whether the lack of that particular annotation reflects that their function is really not associated to pancreas development at all, or if the association to pancreas development or this protein emerged in evolution later than the emergence of the pancreas itself (in level 9 and beyond).

## Discussion

Finding the evolutionary history of a protein can be facilitated by the selection of homologs in distant species. ProteinPathTracker uses a stepwise strategy to provide a selection of aligned protein homologs in species at increasing taxonomic distances from a central species, towards a taxon including a second distant species. Searches between species are performed with the specific goal of providing a chain of orthologs related to the protein used as query. Homologs are included even if they are not orthologs, but never used to search for another homolog.

In any case, our method tends to find orthologs. To evaluate this, we executed a systematic test in ProteinPathTracker using the default path (cellular organisms-Homo) with the default species and using as input sequences the complete proteome of *Escherichia coli* (the default in the first taxa). We used the proteomes given by the orthology-based method OrthoMCL (release 5) to generate orthologous pairwise relationships using *E. coli* proteins as reference, to allow for comparisons. The test was also done with direct RBHB. PPT could recover 72% of orthologs from OrthoMCL, and up to 79% when considering only the best 25% scoring orthologous pairs in OrthoMCL (Additional file 2: Figure S1). These analyses suggest that PPT overlaps OrthoMCL mostly when it agrees with pure RBHB, and the overlap improves when the situation is simpler. The large number of cases detected by PPT but not by OthoMCL or RBHB, reflects that PPT provides paths for *E. coli* proteins that probably have no orthologs in human, pointing out that PPT offers an alternative in the analysis of the evolution of complex protein families.

Note that the way ProteinPathTracker helps the user is intimately related to the pragmatic choice on a single path. This has the advantage of simplicity, because it is easier to understand a chain of 15 related proteins (pairwise aligned) connecting two distant species instead of a multiple sequence alignment (MSA) of hundreds of proteins of a family. We admit that the MSA contains more information, but the interpretation of the family by focusing on a particular species and inferring ancestral versions towards an ancestor common to a distant species, is advantageous to understand the relations between sequence and function in the protein family and their evolution.

Although the choice of path and species is pragmatic, the speed of the method allows to explore other paths, species chosen to represent it, and regions of the protein. This can be crucial to support or reject different homologs. Ultimately, the user can collect the different sequences, align them and apply different phylogenetic analyses. But at least our method could be expected to help directing the choice of species to be included in the analyses and give hints about the evolutionary history of the family.

The current version of ProteinPathTracker allows to study six evolutionary pathways. We chose these according to our experience in protein family analyses and data availability. We are certainly aware that other researchers might be interested in different evolutionary paths but considering all possible ones is outside our capacity. To account for this, our web site encourages users to provide us with feedback, which includes requesting the inclusion of other paths and genomes.

## Conclusions

The study of the evolutionary history of a protein can be facilitated by the selection of homologous sequences in other species. The search for these homologous sequences is usually a time-consuming process, which mainly depends on the sequence similarity between proteins given by the evolutionary distance between two organisms. The task of assessing a comprehensive evolutionary path of a given protein requires homology searches between distant species and is likely to involve trial and error. This procedure is time consuming and depends on user knowledge. ProteinPathTracker was built to automatise such process and allows quick navigation through the evolution of a protein in a controlled way, via the selection of a customisable evolutionary path in multiple taxa from a variety of six different paths. Its simplicity is tightly connected to being the automation of a common practice, meant to be brought closer to the needs of non-expert users.

Once the homologous sequences are located along the selected evolutionary path, additional optional functionality of the web tool allows the user to follow the evolution of a subsequence within them. For example, to study the evolution of a protein domain in a multidomain protein we can lock this domain of interest in the found sequences.

The GO term annotations of each of the homologs provide supplementary information to help assessing the evolution in function or subcellular location of the protein of interest throughout its evolution. Furthermore, the comprehensive analysis of GO terms within specific taxa hints at the time of origin of proteins related to specific functions or biological processes.

Overall, ProteinPathTracker is an easy-to-use web tool that automatises the practice of looking for homologs in distant species in a straightforward way for non-expert users. The additional optional functionalities use the found sequences to extract additional information from them. In conclusion, we believe that ProteinPathTracker provides a solution to the time-consuming process of a step-by-step search for homologous sequences to evaluate the evolutionary history of a protein.

## Methods

### Data retrieval

A set of 193 complete proteomes was downloaded from the Proteomes section in UniProt database release 2017_03 [31]. For quality, reference proteomes of completely sequenced model organisms were selected. An additional restraint was that the species were distributed along six distinct evolutionary paths, belonging to different taxa. They are placed in the last common taxon with the terminal node of the path. The six evolutionary paths are: 1) cellular organisms – Homo (default path), 2) Primates – Homo, 3) Viridiplantae – Arabidopsis, 4) Fungi – Schizosaccharomyces, 5) Bacteria – Escherichia, and 6) Arthropoda – Drosophila. The full list of proteomes is available in Additional file 1.

Annotations for each protein were obtained from their UniProt entries.

### Locking a region to follow its evolution

To map the evolutionary conservation of a subsequence throughout the protein path in evolution, such region can be ‘locked’ by providing its coordinates in the query sequence. The alignments obtained from the BLAST reports are used to assess if the subsequence is conserved in the ortholog/homolog or not. This functionality is not selected by default. The length of the subsequence must be in the interval 10–100 amino acids.

When an orthologous sequence is found, ProteinPathTracker tries to locate the locked region, requiring a threshold of sequence coverage (75% by default). The coordinates of the last mapped locked region are used to discover the subsequence. We can also rescue a region that was lost in any of the orthologs; for example, if one protein is a fragment and lacks the locked region, it can still be mapped in the following orthologs because the coordinates in the query protein will be used.

As the locked region is ultimately mapped using the coordinates in the query, ProteinPathTracker yields different results depending on the query sequence used for each execution. The locked region may be changed iteratively after an execution by selecting a query amongst the list of orthologs and a new set of coordinates in it.

To ease the interpretation of the results, a logo built out of the locked regions is provided (using the source code from WebLogo version 2.8.2) [32].

### Prevalence of GO terms in the data

To illustrate general evolutionary principles that can be obtained with a systematic use of ProteinPathTracker, we analysed all human proteins with it and compared the GO annotations of the proteins found at each taxonomic distance. Using the default path (cellular organisms – Homo) and a proteome per taxon (the default proteomes, see Additional file 1 for details), we executed ProteinPathTracker with default parameters using all Swiss-Prot database entries from the human proteome as query sequences (20188 proteins). We computed the presence of orthologs/homologs from the query in the default proteomes and associated this information with the GO terms of each protein. The 15 taxa used in the default path are: 0) cellular organisms, 1) Eukaryota, 2) Opisthokonta, 3) Metazoa, 4) Bilateria, 5) Deuterostomia, 6) Chordata, 7) Osteichthyes, 8) Sarcopterygii, 9) Tetrapoda, 10) Amniota, 11) Mammalia, 12) Eutheria, 13) Primates, 14) Homo.

### Data availability

The datasets generated during the current study are available from the corresponding author on reasonable request.

## Additional files

Additional file 1: List of complete reference proteomes used in the web tool, organised by evolutionary path. (XLSX 13 kb)

Additional file 2: **Figure S1.** Number of orthology pairwise relationships calculated with OrthoMCL, ProteinPathTracker and Reciprocal Best Hit Blast (RBHB) in 15 species, using the proteomes provided by OrthoMCL in the default species from the default path in ProteinPathTracker, and taking *E. coli* proteins as reference. a) All OrthoMCL pairs. b) Only the best 25% scored OrthoMCL pairs. (PNG 388 kb)

## Abbreviation

RBHB: Reciprocal Best Hits BLAST
