# Supplementary figures and images for: Automated selection of homologs to track the evolutionary history of proteins
Source: BMC Bioinformatics. 2018 Nov 19;19:431. doi: 10.1186/s12859-018-2457-y (PMC6245638; doi:10.1186/s12859-018-2457-y)

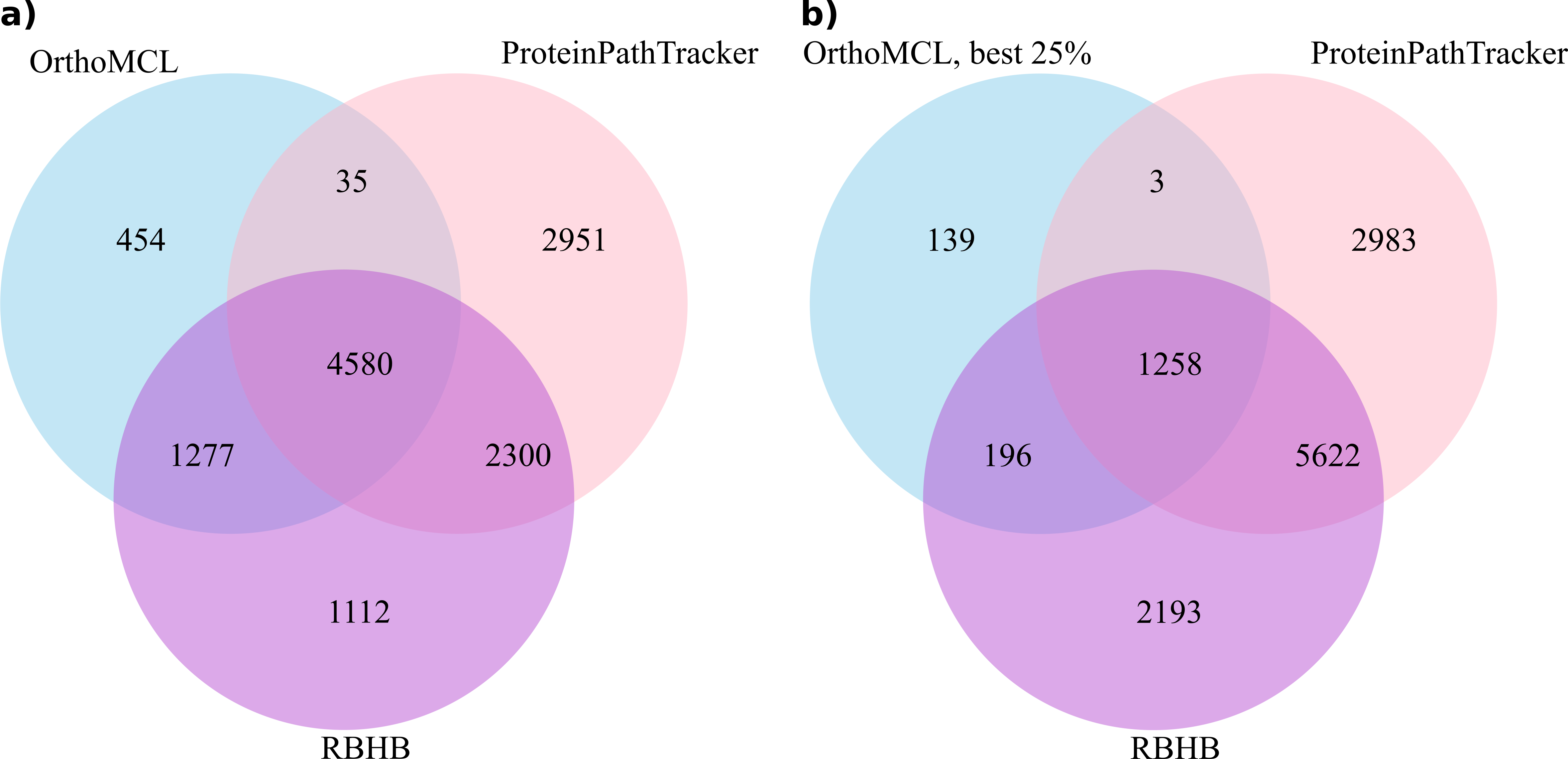

Supplement: Supplementary file 2 — Figure S1. Number of orthology pairwise relationships calculated with OrthoMCL, ProteinPathTracker and Reciprocal Best Hit Blast (RBHB) in 15 species, using the proteomes provided by OrthoMCL in the default species from the default path in ProteinPathTracker, and taking E. coli proteins as reference. a) All OrthoMCL pairs. b) Only the best 25% scored OrthoMCL pairs. (PNG 388 kb) [file 12859_2018_2457_MOESM2_ESM.png]
